# Biosorption of Cr(VI) by *Theobroma cacao* pericarp

**DOI:** 10.1007/s11356-024-34971-7

**Published:** 2024-10-04

**Authors:** Miguel Fernández-Pezua, Carmencita Lavado-Meza, Leonel De la Cruz-Cerrón, Francisco Gamarra-Gómez, Elisban Sacari-Sacari, Carmen Lavado-Puente, Juan Z. Dávalos-Prado

**Affiliations:** 1 Faculty of Engineering, Professional School of Environmental Engineering, Universidad Nacional Intercultural de la Selva Central Juan Santos Atahualpa (UNISCJSA), La Merced, Chanchamayo, 1285 Peru; 2 Faculty of Engineering, Universidad Continental (UC), Huancayo, 12004 Peru; 3 Nanotechnology Laboratory, Faculty of Engineering, Universidad Nacional Jorge Basadre Grohmann (UNJBG), Tacna, 23001 Peru; 4 Instituto de Química Física “Blas Cabrera”, CSIC, 28006 Madrid, Spain

**Keywords:** Cr(VI) biosorption, *Theobroma cacao*, Agricultural waste, Langmuir isotherms, Heavy metals

## Abstract

This paper reports a comprehensive study of *Theobroma cacao* pericarp (TCP) residues, which has been prepared, characterized, and tested as an inexpensive and efficient biosorbent of Cr(VI) from aqueous solutions. The maximum adsorption capacity of TCP obtained at optimal conditions (pH = 2, dose = 0.5 g L^−1^, *C*_0_ = 100 mg L^−1^) was *q*_max_ = 48.5 mg g^−1^, which is one of the highest values reported by the literature. Structural and morphological characterization has been performed by FTIR, SEM/EDX, and pH_PZC_ measurements. FTIR analysis revealed the presence of O–H, –NH, –NH_2_, C = H, C = O, C = C, C–O, and C–C functional groups that would be involved in the Cr(VI) biosorption processes. The experimental equilibrium data of biosorption process were successfully fitted to non-linear Langmuir (*R*^2^ = 0.95, *χ*^2^ = 11.0), Freundlich (*R*^2^ = 0.93, *χ*^2^ = 14.8), and Temkin (*R*^2^ = 0.93, *χ*^2^ = 14.7) isotherm models. Kinetics experimental data were well adjustment to non-linear pseudo-2nd (*R*^2^ = 0.99, *χ*^2^ = 2.08)- and pseudo-1st-order kinetic models (*R*^2^ = 0.98, *χ*^2^ = 2.25) and also to intra-particle Weber-Morris (*R*^2^ = 0.98) and liquid film diffusion (*R*^2^ = 0.99) models. These results indicate that Cr(VI) biosorption on heterogeneous surfaces as well as on monolayers of TCP would be a complex process controlled by chemisorption and physisorption mechanisms. The thermodynamic results indicate that the Cr(VI) biosorption on TCP is a feasible, spontaneous, and endothermic process. TCP can be regenerated with NaOH and reused up to 3 times.

## Introduction

In many places of the world, population growth and uncontrolled industrialization generate wastewater, whose discharges with poor or no treatment contaminate water resources (Quitian 2021). Wastewater contains several contaminants, among them heavy metals (Soon et al. 2022; Pérez-Marín et al. 2024) that can pass not only into the food chain but also into the atmosphere. These contaminants (such as Cr, Hg, Cd, and Pb) represent one of the most pressing global problems, due to their high degree of toxicity and persistence in ecosystems, causing negative effects on the health of the population and the environmental (Murga and González 2020; Tejada-Tovar et al. 2020).

Chromium (Cr) is a heavy metal found in wastewater in both trivalent (III) and hexavalent (VI) forms (Quitian 2021); the latter being the most toxic, due to its high solubility and mobility in biological systems (Mishra et al. 2020), while the trivalent form (III) is rather considered a micronutrient with important functions in the metabolism of sugars and lipids (Oliveira 2012). Cr(VI) is used in the fabric factory, metal finishing, metallurgy, chrome electroplating, glass manufacturing, wood preservation, dye manufacturing, and tanning (Wang et al. 2023). Exposure to this metal in humans can cause respiratory, gastrointestinal, immunological, hematological, reproductive, dermal, ocular, genetic, and particularly with carcinogenic effects (Wise et al. 2019), sinceCr (VI) and Cd are categorized, according to International Agency for Research on Cancer, as “Group 1” carcinogen. Therefore, Cr(VI) is one of the metallic species that poses a serious environmental problem and has harmful health effects that make its elimination essential.

Currently, there are several techniques for the treatment and removal of Cr (VI) from contaminated aqueous systems: precipitation, redox, ion-exchange, filtration, reverse osmosis, electrochemical treatment, membrane technologies, recovery by evaporation and adsorption, and others (Pabón et al. 2020; Dev et al. 2024). Several of them have disadvantages such as high operational costs, high reagent and energy consumptions, and low selectivity and in some cases can be counterproductive, because can yield polluting sludge (Pari 2020). The adsorption process is considered an effective method for the removal of numerous pollutants due to the high efficiency, low operating cost, ease of operation, good selectivity, availability, and sustainability of the process. However, the high cost of conventional and commercial adsorbents, including those with activated-carbon, is a disadvantage that drives the search for new economical, highly effective, reusable, and environmentally friendly adsorbents capable of replacing conventional and commercial ones. Biological techniques, based on biosorption, bioreduction, and bioaccumulation processes are considered as economical and environmentally beneficial techniques (Xie 2024). Particularly, agricultural materials such as agro-industrial waste materials show potential biosorption capacity for various pollutants (Bhatnagar and Sillanpää 2010). So, these biosorbent materials constitute an effective, economic, and ecological alternative for the removal heavy metals such as Cr(VI) from aqueous solutions, due to their structural composition, availability in abundance, renewable nature, and low cost (Miranda 2019). The composition of agro-industrial waste materials includes lignocellulosic compounds (cellulose, hemicellulose, and lignin) containing a variety of polar functional groups (–OH, –COOR, –CO, etc.) that enhance their metallic adsorptive capacities (Pérez et al. 2020; Dev et al. 2024). The literature reports studies of a wide variety of agricultural by-products on Cr(VI) biosorption employing, e.g., olive leaves *Chemlali* pruning waste (Rzig et al. 2024), *Phoenix dactylifera* coir wastes (Rambabu et al. 2021), coffee grounds (Silva 2021), banana peel (Pari 2020), eucalyptus leaves (Miranda 2019), water hyacinth (Tejada et al. 2020), *Sargassum tenerrimum* (Bazzazzadeh et al. 2020), gliricidia leaf (Suganya et al. 2019), *Colocasia esculenta* leaves (Nakkeeran et al. 2016), and Teff straw (Tadesse et al. 2014).

It is important to mention that the search and evaluation of lignocellulosic biosorbents for Cr(VI) removal is a promising area of research (Dev et al. 2024), and the potential use of chemically modified lignocellulosic wastes, as efficient and low-cost biosorbents (Jamshaid et al. 2017; Nigam et al. 2019; Zhang et al. 2020), for large-scale removal of heavy metal ions from wastewater, is an interesting environmental challenge.

In Peru, the Junín zone is one of the regions with the highest cocoa production (29,774 T year^−1^). The process of obtaining cocoa beans generates large quantities of waste, called pericarps (MIDAGRI 2022), which if not properly treated can become an environmental problem (Sanchez 2018; Vásquez et al. 2019). It is known that the cocoa pericarp contains cellulose, hemicellulose, lignin, and ash that favor the adsorption of Cr (VI) (Sánchez 2016; Pérez et al. 2020) and also of Pb(II) and Cd(II) (Lavado-Meza et al. 2023a, c). Therefore, this material is promising since it may be susceptible to suitable chemical modifications to be used as a low-cost and highly effective biosorbent for removing various heavy metals from wastewater.

In this context, the goals of this work are the following: (i) Prepare *Theobroma cacao* pericarp agro-industrial waste (TCP) collected from Junin-Peru region. (ii) Characterize TCP, regarding the morphology and surface structure by means SEM/EDX, FTIR, and pH_PZC_ measurements. (iii) Evaluate the parameters (dose, pH, and initial Cr(VI) concentration) that affect the biosorption capacities of TCP to remove Cr(VI). (iv) Study the kinetic, thermodynamic, and mechanisms of the biosorption process.

## Methods and materials

### Preparation of TCP biosorbent

The preparation of the biosorbent was carried out according to the procedures described by Pari (2020) and Lavado-Meza et al. (2023b). *Theobroma cacao* pericarp waste (TCP) was collected from Alto Huacará (Chanchamayo province) located at Junín region in Peru. It was washed with plenty of deionized water, dried at 70 °C for 48 h. After that, the dried sample was ground and sieved using a 70 mesh.

### Characterization of TCP biosorbent

Point of Zero Charge pH (pH_PZC_) evaluation was carried out following the procedure described by Lavado-Meza et al. (2023b): It was prepared a mixture of 1 g of TCP biosorbent with 50 mL of aqueous solutions at different initial pHs (pH_0_) ranging from 2.0 to 9.0. The acid dilutions were prepared from 1 M HNO_3_ solution, while the basic dilutions from 1 M NaOH. After 24 h of equilibrium, the final pHs (pH_f_) were measurements by means a potentiometer (HANNA HI1271 CHECKER). pH_PZC_ was determined from the intersection of ΔpH (= pH_0_ − pH_f_) curve with pH_0_ axis.

Fourier transform infra-red spectrophotometer (FTIR, Bruker Invenio R/Platium ATR, Ettlingen, Germany) was used to identify, over a range of 4000 to 500 cm^−1^, the functional groups present on the surface of TCP, before and after Cr adsorption. The morphology and elemental analysis of these surfaces were also performed using scanning electron microscopy, coupled with energy dispersive X-rays (SEM/EDX, Thermo Scientific Co, Eindhoven, The Netherlands).

### Biosorption experiments

Batch experiments were carried out using K_2_Cr_2_O_7_ solution, varying Cr(VI) concentrations between 10 and 150 mg L^−1^. The dose of TCP biosorbent was varied in the range of 0.5 a 3 g L^−1^ and adjusted to a pH in the range of 2.0 to 6.0 by adding 0.01 M HNO_3_ or 0.01 M NaOH. The solutions, at room temperature, were stirred at 300 rpm for *t* = 120 min, and the samples were extracted and filtered at certain time intervals. The Cr(VI) concentrations, before and after adsorption, were evaluated following ASTM D1687–02 (2007), where Cr(VI) reacts with diphenylcarbazide in an acidic medium to produce a reddish purple color, which intensity—proportional to the concentration of Cr(VI)—is measurement using UV/VIS spectrophotometer (UV-1900i, UV visible spectrophotometer, Shimadzu, Japan) at the maximum absorption wavelength (*λ*_max_, 540 nm). All biosorption experiments were replicated three times. Both, adsorption capacity *q*_*e*_ (mg·g^−1^) and removal efficiency %RE, were calculated by using Eqs. (1) and (2), respectively (Araújo et al. 2018; Lavado-Meza et al. 2023c).

1 $${q}_{e}=\frac{({C}_{0}-{C}_{e})}{M}\times V$$

2 $$\%\text{RE}=\frac{({C}_{0}-{C}_{e})}{{C}_{0}}\times 100$$

where *C*_0_ and *C*_*e*_ (mg L^−1^) are initial and equilibrium final Cr(VI) concentrations, respectively; *V* (in L) is the volume of solution; and *M* (in g) is the TCP biosorbent mass.

Both isothermal-adsorption and kinetic experimental data were adjusted to the different models described in Table 1. Biosorption thermodynamic functions, such as standard Gibbs energy (∆*G*^0^), enthalpy (∆*H*^0^), and entropy changes (∆*S*^0^), were evaluated using the Eqs. (3) and (4) (Van’t Hoff equation) (Mosaffa et al. 2024a):

3 $$\Delta {G}^{0}=-RT\text{ln}({K}_{c})$$

4 $$\text{ln}({K}_{c})=\frac{\Delta {S}^{0}}{R}-\frac{\Delta {H}^{0}}{RT}$$

where *K*_*c*_ is the equilibrium constant, *K*_*c*_ = *C*_es_*/C*_e_; *C*_es_ and *C*_e_ are the equilibrium Cr(VI) concentrations in the biosorbent and in the solution, respectively. *R* is the universal gas constant, and *T* (in K) is the temperature of the solution.

**Table 1:** Models used for the Cr(VI) biosorption onto TCP

Model	Equation	Parameters	References
Isotherm adsorption models			
Langmuir	${q}_{e}={q}_{\text{max}}\frac{{K}_{L}{C}_{e}}{1+{K}_{L}{C}_{e}}$ ${R}_{L}=\frac{1}{1+{K}_{L}{C}_{e}}$	*q*_*e*_: adsorption capacity at equilibrium (mg g^−1^) *q*_max_: maximum adsorption capacity (mg g^−1^) *K*_L_: Langmuir constant related to the affinity between sorbent and sorbate (L mg^−1^) *C*_*e*_: adsorbate concentration at equilibrium (mg L^−1^) *R*_*L*_: Separation factor	(Araújo et al. 2018; Mzinyane et al. 2023; Mosaffa et al. 2024b)
Freundlich	${q}_{e}={K}_{\text{F}}{{C}_{e}}^{{~}^{1}\!\left/ \!{~}_{n}\right.}$	*K*_F_: equilibrium constant (mg g^−1^) (L mg)^1/n^ *n*: constant related to the affinity between sorbent and sorbate	
Temkin	${q}_{e}=\frac{RT}{B}\text{ln}({A}_{\text{T }}{C}_{e })$	*R*: universal gas constant (J mol^−1^ K^−1^) *T*: temperature (K) *A*_T_: Temkin’s equilibrium constant (L mg^−1^) *B*: adsorption energy variation (J mol^−1^)	
Kinetic models			
Pseudo-1st order	${q}_{t}={q}_{e}\left(1-{e}^{{-k}_{1}t}\right)$	*q*_*e*_: adsorption capacity at equilibrium (mg g^−1^) *q*_*t*_: amount of Cr(VI) retained per unit mass of biosorbent at time *t* (mg g^−1^) *k*_1_: the first-order kinetic constant (1 min^−1^)	(Mzinyane et al. 2023; Mosaffa et al. 2024b)
Pseudo-2nd order	${q}_{t}=\frac{{{{q}_{e}}^{2}k}_{2}.t}{1+{q}_{e}.{k}_{2}.t}$	*k*_2_: rate constant adsorption (g mg^−1^ min^−1^)	
Weber Morris intra-particle diffusion	${q}_{t}={k}_{\text{id}}.{t}^{0.5}+C$	*k*_*id*_: intraparticle diffusion rate constant (mg g^−1^ min^−1/2^) *C*: constant	
Liquid film diffusion	$\text{ln}\left(1-\frac{{q}_{t}}{{q}_{e}}\right)=-{k}_{\text{fd}}.\text{t}+C$	*k*_fd_: film-diffusion rate coefficient (min^−1^) *C*: constant	

### Desorption-regeneration experiments

Desorption or regeneration experiments were carried out using 0.1 M NaOH and water distilled as eluents. 0.5 g of TCP previously loaded with Cr(VI) under optimal conditions (pH 2, *C*_0_ = 100 mg L^−1^) then filtered, washed, and dried at 80 °C for 3 h was subjected to desorption process by adding 25 mL of each eluent and stirring at 100 rpm for 1 h. After that, the regenerated TCP was rinsed with distilled water many times before being dried and reused again. The desorption or regeneration efficiency (%*D*) of TCP was determined using Eq. (5) (Lavado-Meza et al. 2023c).

5 $$\%\mathrm D\;=\;\frac{\mathrm{Cr}\;\left(\mathrm{VI}\right)\;\mathrm{desorbed}\;\mathrm{from}\;\mathrm{biomass}}{\mathrm{Cr}\;\left(\mathrm{VI}\right)\;\mathrm{adsorbed}\;\mathrm{on}\;\mathrm{biomass}}\;\times\;100$$

## Results and discussion

### Biosorbent properties, pH_PZC_measurement, and pH effects

**Fig. 1:**
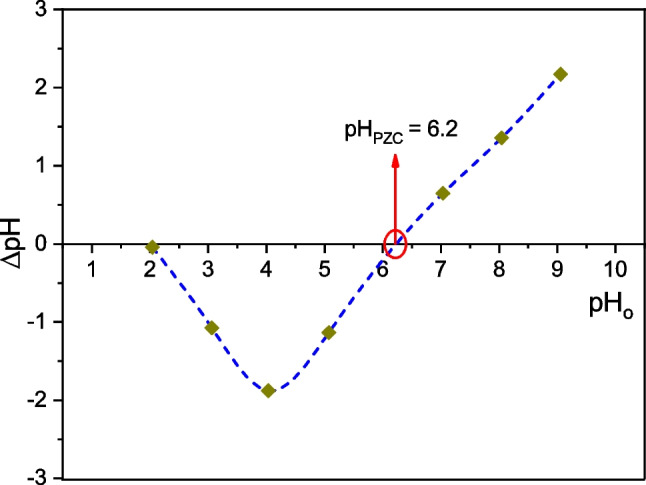
Evaluation of pH_PZC_ value for the TCP biosorbent

In Fig. 1, the ΔpH (= pH_0_ − pH_f_) vs. pH_0_ curve is depicted, where can be seen the pH_PZC_ value equal to 6.2. It indicates that the surface of the TCP is positively charged at pH < 6.2 or negatively charged at pH > 6.2. For the 1st case, the TCP surface favors the electrostatic attraction of anions, while for the 2nd case, favors the attraction of cations. Our pH_PZC_ value is very close to that reported by Sánchez (2016) (pH_PZC_ = 6.9) and Machado (2017) (pH_PZC_ = 7.0) for cocoa-pericarp biosorbents.

**Fig. 2:**
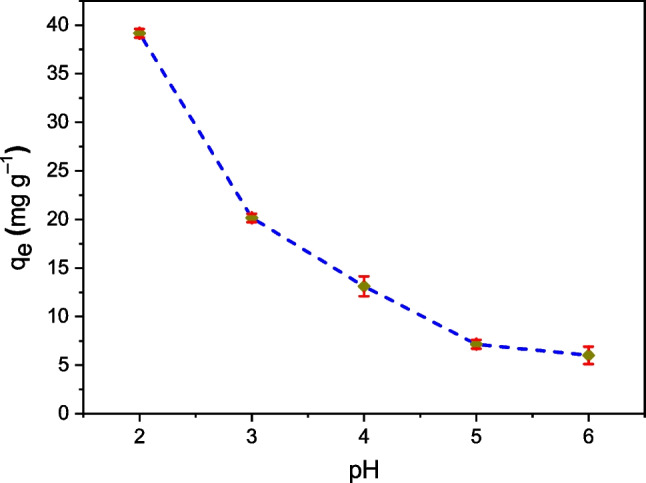
pH influence on the Cr(VI) adsorption capacity q_e_ of TCP. Experimental conditions: *T* = 20 °C, sorption or contact time *t* = 120 min, dose = 0.05 g L^−1^, *C*_0_ = 100 mg L^−1^

Figure 2 shows the pH influence on the Cr (VI) adsorption capacity (*q*_*e*_) of the TCP. It can be appreciated that *q*_*e*_ decreases with increasing pH, being the highest value at pH 2. At this pH, and according to the value of pH_PZC_ = 6.2, the surface of the TCP biosorbent would be positively charged due to the protonation of the active sites (formed by amino and carboxyl groups) which would strongly attract the anions of Cr (VI) such as HCrO_4_^–^ and Cr_2_O_7_^–2^ (Mondal et al. 2019; Ren et al. 2022). Increases in pH would induce a less positive biosorbent surface that would decrease the adsorption of Cr(VI) anions (Blázquez et al. 2014; Mondal et al. 2019; Li et al. 2021; Narayanasamy et al. 2022; Ren et al. 2022). A more detailed explanation was given by Park et al. (2005), Verma et al. (2021), and Pertile et al. (2021) who included reduction process of Cr(VI) to Cr(III) following one of two proposed mechanisms: (1) Cr (VI) is directly reduced to Cr (III) in aqueous phase by contact with the electron donating groups of the biosorbent; (2) previous anionic adsorption of Cr (VI), this is reduced to Cr(III) through electron-donating groups of the biosorbent, and then, Cr (III) ions are released by electrostatic repulsion with the positive biosorbent surface.

These results are comparable to those obtained for waste biosorbents such as eucalyptus(Miranda 2019), banana-peel (Pari 2020), coffee-bora (Silva 2021), gliricidia-leaf (Suganya et al. 2019), and water-hyacinth (Tejada et al. 2020), where at pH < 2.5 are reported high values of Cr (VI) adsorption capacities, *q*_*e*_.

**Fig. 3:**
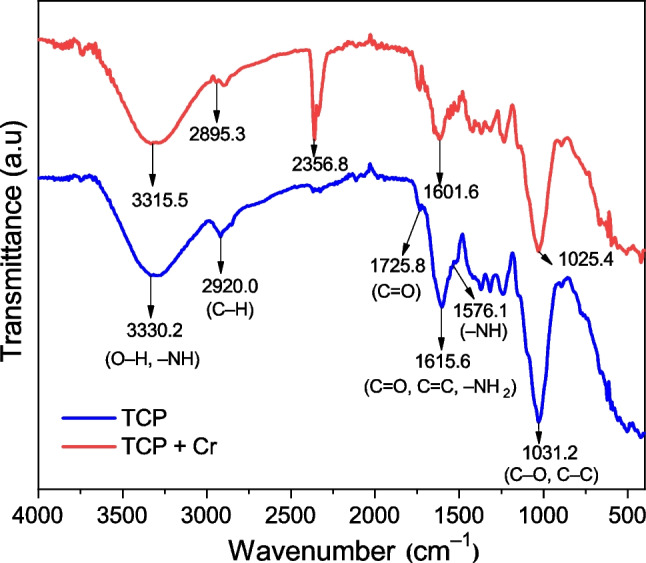
FTIR spectra before (blue) and after (red) Cd(VI) adsorption on TCP, at pH 2, *C*_0_ = 100 mg L^−1^, *T* = 293 K, *t*_sp_ = 120 min, dose = 0.5 g L^−1^

FTIR spectral analysis was done to identify functional groups present on the surface of TCP. Figure 3 shows typical FTIR spectra of TCP before and after Cr(VI) adsorption. The FTIR spectra of the unloaded-Cr samples show band positions at (i) 3330.2 cm^−1^ assignable to O–H and N–H bond stretching vibrations in cellulose, hemicellulose, lignin, and pectin (Olu-owolabi 2012; Lavado-Meza et al. 2023b); (ii) 2920.0 cm^−1^, attributed to stretching vibrations of C–H bonds in aliphatic acids and methyl and methylene groups (Olu-owolabi 2012; Rzig et al. 2021); (iii) 1725.8 cm^−1^, would correspond to the C = O stretching vibrations of the hemicelluloses (Rzig et al. 2021); (iv) 1615.6 cm^−1^, related to stretching vibrations of C = O in ketones and C = C bonds in carbonyl groups present in lignin (Pérez et al. 2020; Nursiah et al. 2023; Mosaffa et al. 2024b); (v) range of 1650 to1450 cm^−1^, would be related to the –NH_2_ scissoring and –NH deformation bands (Suganya et al. 2019; Ren et al. 2022); (vi) 1031.2 cm^−1^, attributed to stretching vibrations of C–OH in alcohols (Olu-owolabi 2012; Mosaffa et al. 2024a) and C–C bonds of carboxylic acids of polysaccharides (Pérez et al. 2020).

After biosorption, the (TCP + Cr) spectrum (Fig. 3) shows changes in the intensity and position of the most important bands described above. Thus, the bands move toward lower values (red-shifting) between 5.8 and 24.7 cm^−1^. These changes reflect the interaction of Cr(VI) with the active sites of TCP biosorbent (Pérez et al. 2020; Rzig et al. 2021; Lavado-Meza et al. 2023a). The intense band at 2356.8 cm^−1^ would correspond to CO_2_ present in the solution (Gök et al. 2022; Ni et al. 2023).

**Fig. 4:**
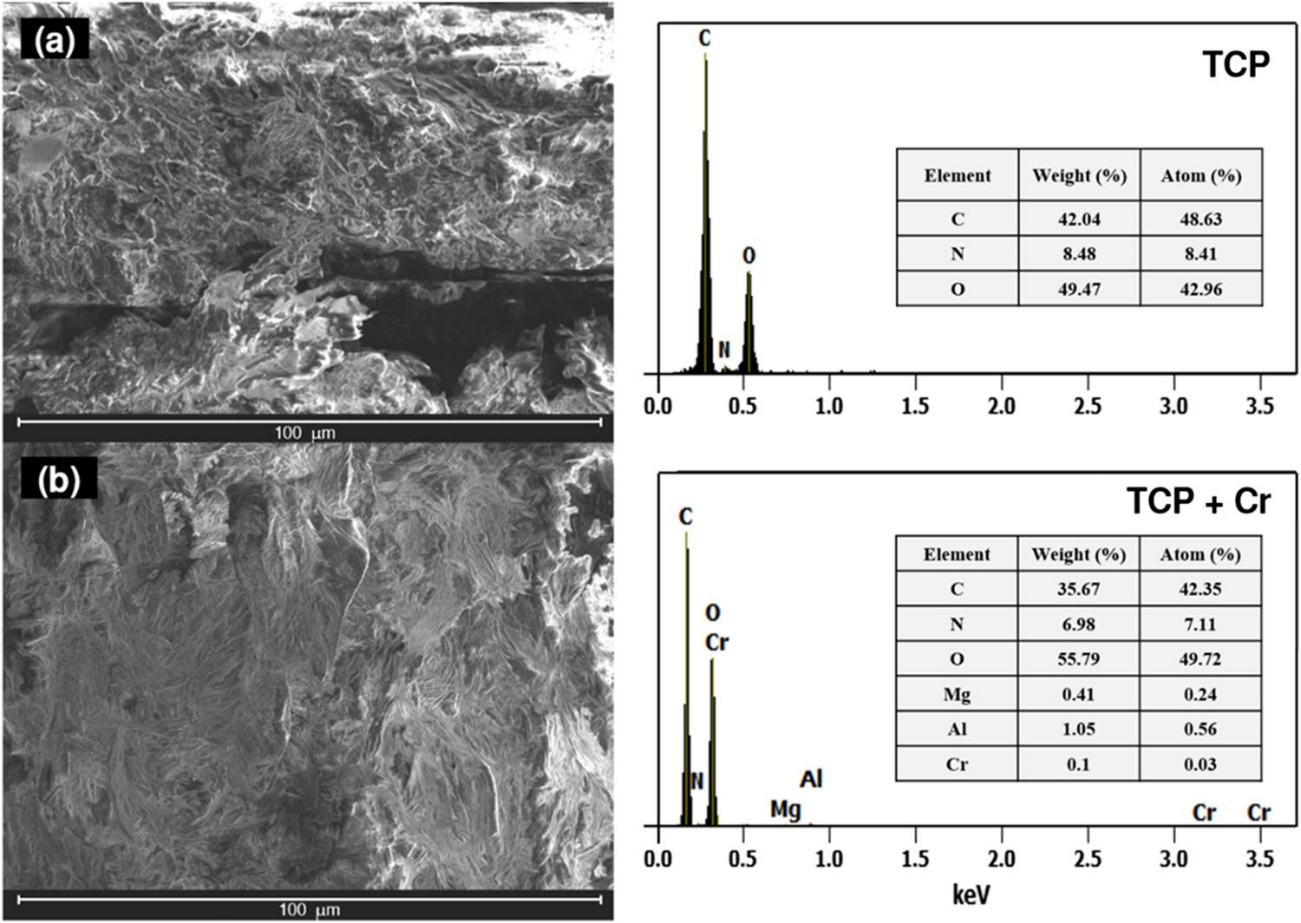
Typical SEM morphology (left) and EDX analysis (right) of TCP, **a** before and **b** after Cr(VI) adsorption. pH 2, *C*_0_ = 100 mg L^−1^, dose = 0.5 g L^−1^

Figure 4 shows SEM micrographs and EDX analysis of the TCP before and after Cr(VI) sorption. TCP clean (Fig. 4a) shows a cracked surface with heterogeneous plates involving an irregular micro-rough structure that would facilitate the adsorption of Cr(VI) (Sanchez 2018).The morphology of the surface after Cr(VI) adsorption is different to clean surface, given that it is more compact, less rough, and heterogeneous (Fig. 4b). The EDX analysis of clean TCP shows C (48.63%), N (8.41%), and O (42.96%) peaks related to its lignocellulosic nature, while TCP loaded with Cr(VI) (TCP + Cr) shows Cr peak in addition to those mentioned, which is evidence of Cr (0.03%) adsorption on the TCP surface. Banchhor et al. (2021) reported similar results after loading with Cr(VI) the biomass of *Simarouba glauca* leaves.

### Biomass dose effect

**Fig. 5:**
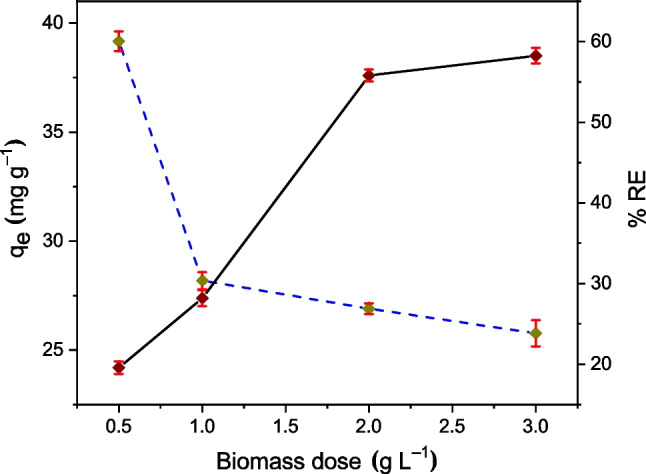
Biomass dose of TCP vs. adsorption capacity *q*_*e*_ (dotted line) and removal efficiency %RE (continuous line) of Cr(VI) at pH 2, *C*_0_ = 100 mg L^−1^, *T* = 20 °C and sorption time *t*_sp_ = 180 min

The dose of biosorbent has a significant impact on both, the equilibrium adsorption capacity (*q*_*e*_) and removal efficiency (%RE) of Cr(VI). The effect of TCP doses, in the 0.5 to 3.0 g L^−1^ range, was investigated at *C*_0_ = 100 mg L^−1^, pH 2, *T* = 20 °C, and sorption time *t*_sp_ = 180 min. The results of *q*_*e*_ and %RE are shown in Fig. 5. It can be appreciated that *q*_*e*_ decreases with an increasing biosorbent dose. In contrast, %RE increases. With an initial dose of 0.5 g L^−1^, the maximum *q*_*e*_ value of 39.2 mg g^−1^ is obtained. These observations can be explained by the availability of active sites on the surface of the biosorbent. At low doses, there is a high concentration of Cr(VI) per unit mass of TCP, leading to rapid saturation of the active sites and higher *q*_*e*_. However, at higher doses, there are more available but unsaturated sites, resulting in a lower *q*_*e*_ but higher %RE due to the increased contact area. Similar results of (*q*_*e*_ vs. biomass dose) were reported by Suganya et al. (2019) and Li et al. (2021) using, respectively, Gliricidia-sepium and lychee peel biosorbents to remove Cr(VI). Then, and according to these authors, the minimum dose (= 0.5 g L^−1^) was considered as the most appropriate amount of TCP for optimal adsorption of Cr(VI).

### Biosorption isotherm

**Fig. 6:**
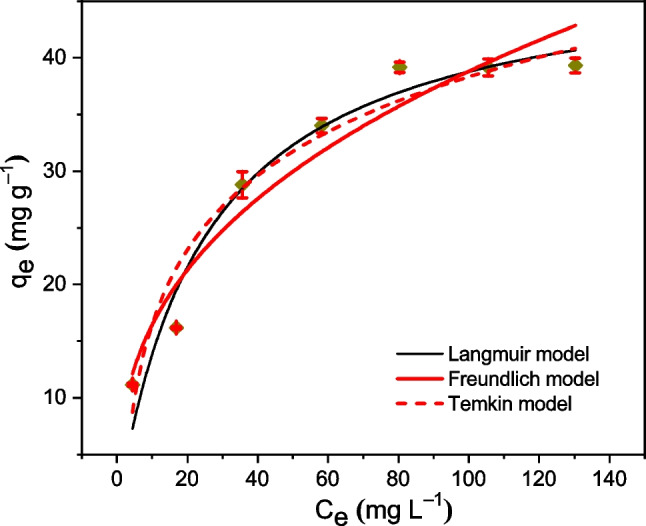
*q*_*e*_ vs. *C*_*e*_ biosorption isotherm. TCP dose = 0.5 g L^−^^1^, *t*_sp_ = 120 min, *T* = 20 °C, pH 2

Cr(VI) biosorption isotherm (*q*_*e*_ vs. *C*_*e*_) was studied, under equilibrium optimal-conditions, in a range of initial Cr(VI) concentrations *C*_0_ between 10 and 150 mg L^−1^, at pH 2, dose = 0.5 g L^−1^ and contact time *t*_sp_ = 120 min. It can be seen that *q*_*e*_ increases with the increase of *C*_*e*_, reaching highest experimental value (39.2 mg g^−1^) for *C*_*e*_ > 80 mg L^−1^ (see Fig. 6). This behavior can be attributed to the interaction of Cr(VI) cations with biosorbent surface. So, at lower Cr(VI) concentrations, reduced competition for active binding sites on the TCP adsorbent surface allows more vacant sites to be available, increasing its biosorption capacity *q*_*e*_. However, as the initial *C*_*e*_ increases, excess Cr(VI) cations difficulty finding available binding sites and *q*_*e*_ slows its growth. At C_e_ higher than 80 mg L^–1^, the q_e_ remains constant, indicating the saturation of binding sites on the TCP surface has been reached (Ren et al. 2022). The (*q*_*e*_ vs. *C*_*e*_) experimental data were fitted (non-linear adjustments) to three models, Langmuir, Freundlich, and Temkin (see Fig. 6). The evaluated parameters are consigned in Table 2. The biosorption-isotherm data are well fitted with the three models (*R*^2^ ≥ 0.93, *χ*^2^ ≤ 14.8), although the best fit, with highest correlation coefficient *R*^2^ (= 0.95) and lowest *χ*^2^ (= 11.0), was obtained with Langmuir model. The Langmuir parameters deduced were, a low value *K*_L_ equal to 0.04 L mg^−1^ with maximum adsorption capacity *q*_max_ equal to 48.5 mg g^−1^. The separation factor value *R*_L_ (= 0.45) is in the range of 0 < *R*_L_ < 1, which provides the favorability of the adsorption process. The 1/*n* (= 0.38) value obtained from the Freundlich isotherm, which is a measure of the intensity of biosorption or surface heterogeneity, is less than one (0 < 1/*n* < 1), indicating that the biosorption of Cr(VI) onto TCP is favorable (Mosaffa et al. 2024a). These values would indicate high affinity of Cr(VI) biosorption on monolayers of TCP (Pertile et al. 2021). However, as mentioned above, the acceptable adjustments with the Freundlich and Temkin models indicate that Cr(VI) biosorption would also occur in TCP multilayers of heterogeneous nature (Freundlich model). The adsorption would be predominantly a physisorption process given that Temkin parameter B (= 262.9 J mol^−1^) is less than 8000 J mol^−1^ (Araújo et al. 2018; Moreira et al. 2019; Mahmoud et al. 2020; Hezam et al. 2022), and the Cr(VI) adsorbates would adhere to the TCP through weak van der Waals interactions. Therefore, all these results indicate that in reality the Cr (VI) biosorption on TCP is rather complex processes, where under the experimental conditions, heterogeneous surface as well as monolayer biosorption might coexist.

**Table 2:** Isotherm parameters of Cr(VI) biosorption on TCP

Models	Parameters	
Langmuir	*q*_max_ (mg g^−1^)	48.5 ± 3.5
	*K*_L_ (L mg^−1^)	0.04 ± 0.01
	*R*_L_	0.45
	*R*^2^	0.95
	*χ*^2^	11.0
Freundlich	*K*_F_ (mg g^−1^) (L mg)^1/*n*^	6.9 ± 1.7
	*N*	2.6 ± 0.4
	1/*n*	0.38
	*R*^2^	0.93
	*χ*^2^	14.8
Temkin	*A*_T_ (L g^−1^)	0.6 ± 0.2
	*B* (J mol^−1^)	262.9
	*R*^2^	0.93
	*χ*^2^	14.7

These results are comparable to those reported by Pari (2020), Mondal et al. (2019), Pant et al. (2022), and Daffalla (2023), where the isothermal Cr(VI) adsorptions are well fitted with Langmuir model. In Table 3, the maximum Cr(VI) biosorption capacities, *q*_max_, of different non-treated agro-industrial waste is consigned. The *q*_max_ of TCP is among the highest values, indicating that TCP is a promising biomass to remove Cr(VI) from contaminated aqueous media.

**Table 3:** Maximum Cr(VI) biosorption capacities, *q*_max_, of non-treated biosorbents coming from agroindustrial waste

Biosorbent	*C*_0_ range (mg L^−1^)	pH	*q*_max_ (mg g^−1^)	References
Banana (*Musa acuminata colla*) peel	10–50	2	36.10	Pari (2020)
Litchi shell	50–100	3	25.78	Li et al. (2021)
Agro-waste *Cocos nucifera*	2–6	2	5.95	Kumari et al. (2021)
*Phoenix dactylifera* coir wastes	50–250	2	138.89	Rambabu et al. (2021)
*Sargassum tenerrimum*	5–50	2	37.7	Bazzazzadeh et al. (2020)
Eucalyptus (*Globulus labill*) leaves	5.5–52	3	16.84	Miranda (2019)
Mosambi (*Citrus limetta*) peel	5–30	2	3.62	Mondal et al. (2019)
*Theobroma cacao* shell	2.5–15	6	2.92	Pérez et al. (2020)
*Colocasia esculenta* leaves	20–100	2	47.62	Nakkeeran et al. (2016)
Orange (*Citrus sinensis*) peel	20–100	3	16.66	Tejada et al. (2015)
Leaves of *Tradescantia pallida*	50–200	2	64.67	Sinha et al. (2015)
*Artocarpus heterophyllus* peel	10–150	2	64.47	Saranya et al. (2018)
*Teff* straw	2.5–15	2	3.51	Tadesse et al. (2014)
*Prosopis cineraria* leaf powder	10–60	5	10.046	Singh et al. (2023)
Pine sawdust-cellulose fibers	20–100	6	9.78	Mzinyane et al. (2023)
*Chemlali* olive leaves	10–1000	3.9	21.2–40.3	Rzig et al. (2024)
Orange peels	10–100	2	5.46	Khalfaoui et al. (2024)
*Euryale ferox* Salisbury seed coat	5–25	2	13.64	Goswami et al. (2024)
*Theobroma cacao* pericarp	10–150	2	48.5	This work

### Biosorption kinetics

**Fig. 7:**
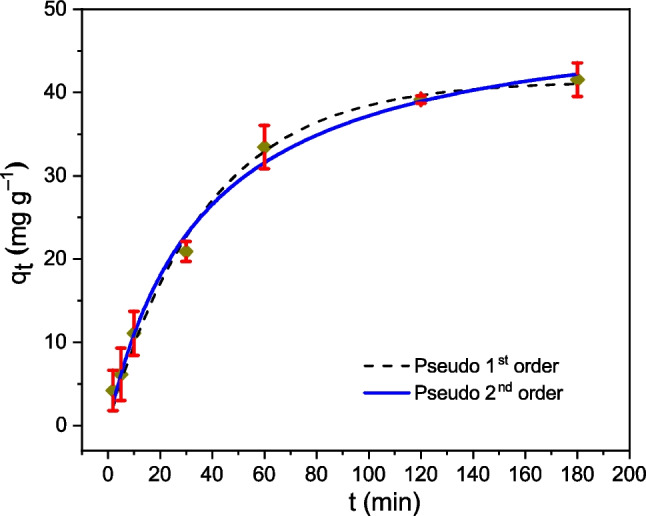
Cr(VI) biosorption kinetics, *q*_*t*_ vs. *t* (contact time). pH 2, dose = 0.5 g L^−1^, *C*_0_ = 100 mg L^−1^, *T* = 20 °C. Pseudo-1st-order (dotted line) and pseudo-2nd-order (solid line) adjustments

**Fig. 8:**
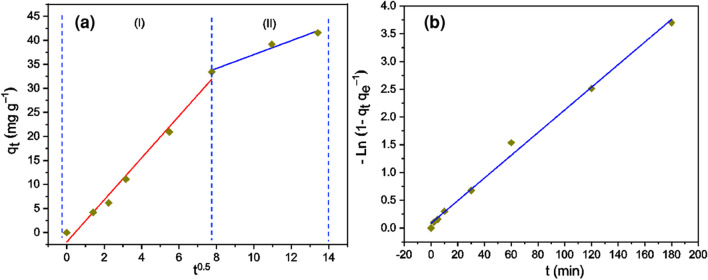
**a** *q*_*t*_ vs. *t*^0.5^ plot fitted with Weber-Morris intra-particle diffusion model; **b** -ln(1 − *q*_*t*_* q*_*e*_^−1^) vs. *t* plot fitted with liquid-film diffusion kinetic model. pH 2, dose = 0.5 g L^−1^, *C*_0_ = 100 mg L^−1^, *T* = 20 °C

The experimental kinetic data were fitted using three adsorption kinetic models (*q*_*t*_ vs. *t*), for both pseudo-1st and pseudo-2nd orders (see Fig. 7) (*q*_*t*_ vs. *t*^1/2^) and [-ln(1 − *q*_*t*_* q*_*e*_^−1^) vs. *t*] for intra-particle diffusion and liquid film diffusion models (see Fig. 8). *q*_*t*_ is the Cr(VI) biosorption capacity or amount of Cr(VI) removed per mass unit of biosorbent, at contact time *t*. The parameters obtained after non-linear adjustments are reported in Table 4. The best fit of (*q*_*t*_ vs. *t*) is obtained with pseudo-2nd-order model (*R*^2^ = 0.99, *χ*^2^ = 2.08), although the pseudo-1st-order model also fits very well (*R*^2^ = 0.98, *χ*^2^ = 2.25). These results would indicate that the Cr(VI) biosorption is a complex process where both chemisorption and physisorption processes take place. According to Park et al. (2005), Verma et al. (2021), and Pertile et al. (2021), the biosorption mechanism would involve anionic adsorption of Cr(VI) and its subsequent reduction to Cr(III) ions due to the presence of electron-donating groups in the biosorbent.

**Table 4:** Kinetic parameters of the Cr(VI) biosorption process

Kinetic models	Kinetic parameters	
Pseudo-1st order	*q*_*e*,cal_ (mg g^−1^)^a^	42.6
	*k*_1_ (1 min^−1^)	0.03
	*R*^2^	0.98
	*χ*^2^	2.25
Pseudo-2nd order	*q*_e,cal_ (mg g^−1^)^a^	41.4
	*k*_2_ (g mg^−1^ min^−1^)	1.19
	*R*^2^	0.99
	*χ*^2^	2.08
Weber-Morris model	*k*_d I_ (mg g^−1^ min^−1/2^)	4.37
	*R*^2^	0.99
	*k*_d II_ (mg g^−1^ min^−1/2^)	1.45
	*R*^2^	0.98
Liquid diffusion model	*k*_fd_ (min^−1^)	0.024
	*R*^2^	0.99

^a^*q*_*e*,cal_ is the calculated adsorption capacity

The (*q*_*t*_ vs. *t*^1/2^) data were fitted with the intra-particle diffusion Weber-Morris model (see Fig. 8a) where two well-defined stages are distinguished: the first stage (0 < *t*^0.5^ < 7.8) shows a rapid growth of *q*_*t*_ at the time *t* (in minutes), which would be associated to a rapid external diffusion of Cr(VI), from aqueous solution to outer surface of the biosorbent (Datt et al. 2022). The second stage (*t*^0.5^ > 7.8) shows a growth of *q*_*t*_(*t*) almost three times slower than in 1st stage, indicating a gradual adsorption process, where Cr(VI) ions would diffuse from the surface into the pores of the biosorbent (Lavado-Meza et al. 2023d). The [-ln(1 − *q*_*t*_* q*_*e*_^−1^) vs. *t*] data were fitted with liquid film diffusion model (see Fig. 8b) which asserts that the rate-of adsorption is governed by adsorbate species transiting through a liquid layer surrounding the solid adsorbent (Mosaffa et al. 2023).

The fit of the data with both diffusion models is excellent (*R*^2^ ≥ 0.98). However, the fitting line does not pass through the origin, showing the incapacity of both Weber-Morris and liquid diffusion models to govern the rate-limiting step of the Cr(VI) biosorption process (Albadarin et al. 2011; Salam et al. 2014; Pant et al. 2022; Al-Odayni et al. 2023). The mechanism based on diffusion via the liquid layer would be applicable at low initial Cr(VI) concentration (Mosaffa et al. 2024a).

### Biosorption thermodynamics

**Fig. 9:**
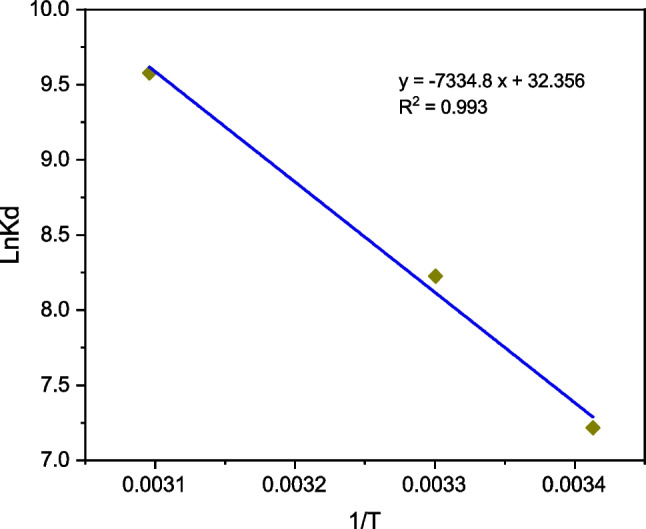
(ln *K*_d_ vs. 1/*T*) plot for Cr(VI) biosorption onto TCP at pH 2, dose = 0.5 g L^−1^, *C*_0_ = 100 mg L^−^^1^

∆*G*^0^(*T*) values were calculated from Eq. (3); while ∆*H*^0^ and ∆*S*^0^ values were deduced from the Van´t Hoff equation fit (Fig. 9). The results are consigned in Table 5 and indicate that the Cr(VI) biosorption process on TCP can be characterized as spontaneous (∆*G*^0^ < 0), endothermic (∆*H*^0^ > 0), with increase in randomness (∆*S*^0^ > 0) of biosorption at the solid–liquid interface. Similar results were found for Cr(VI) biosorption on treated-Juniperus procera leaves (Ali et al. 2019), mesoporous metal–organic structure (Babapour et al. 2022), derived of *Arundo donax stem* (Bhattarai et al. 2022) and agro-waste *Cocos nucifera* (Kumari et al. 2021).

**Table 5:** Thermodynamic parameters of Cr(VI) biosorption on TCP

∆*G*^*0*^ (kJ mol^−1^)			∆*H*^0^ (kJ mol^−1^)	∆*S*^0^ (J mol^−1^ K^−1^)
293 K	303 K	323 K		
− 17.6	− 20.7	− 25.7	61.1	269.0

### Desorption or regeneration of TCP

**Fig. 10:**
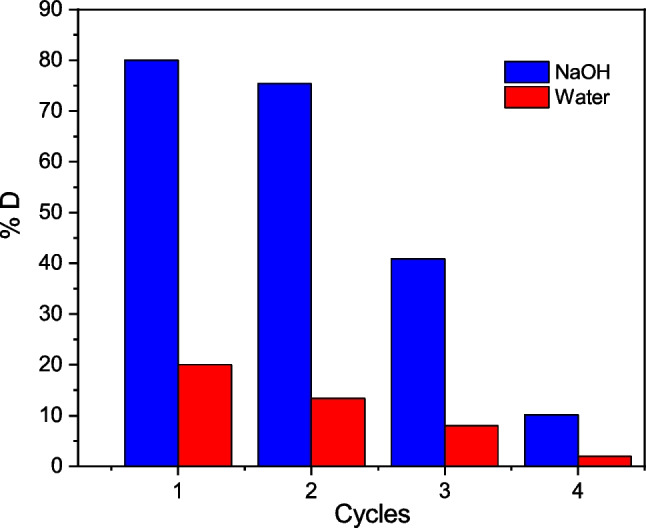
Desorption or regeneration efficiency (%*D*) of TCP biosorbent

The desorption or regeneration efficiency (%*D*) of a biosorbent is a crucial factor influencing the economics of the biosorption process, specifically the operating costs (Mosaffa et al. 2024a). Four desorption or regeneration cycles were carried out to evaluate the %*D* of TCP, using NaOH and distilled water as eluents (see Fig. 10). NaOH was the most efficient desorbent, with %*D* > 40% for the first 3 cycles. On the contrary, %*D* ≤ 20% was achieved with distilled water. The Cr(VI) desorption from the TCP surface in a basic pH environment would be due to a significant exchange among CrO_4_^–2^, dominant Cr(VI) species in alkaline solution, and OH^−^ cations (Rzig et al. 2021), although the regeneration effect of TCP was drastically reduced after the 3rd cycle. Therefore, TCP is a biosorbent with an appreciable reuse performance.

## Conclusions

In the present study, the use of agricultural residues of *Theobroma cacao* pericarp (TCP) as an effective biosorbent of Cr (VI) ions from aqueous solutions was investigated. It has been determined the point of zero charge pH (pH_PZC_) at 6.2. FTIR analysis showed the presence of functional groups such as O–H, C–H, C = O, C = C, C–O, C–C, –NH_2_, and –NH on the TCP surface, which favored the Cr(VI) biosorption process. SEM/EDX analyses showed irregular and rough surface morphologies on TCP clean, which changed to more compact and homogeneous after Cr(VI) adsorption. Optimal Cr(VI) biosorption capacities, *q*_*e*_, were determined at pH 2, biosorbent dose = 0.5 g L^−1^ and initial Cr(VI) concentration *C*_0_ = 100 mg L^−1^. The adsorption isotherm data were successfully fitted with non-linear Langmuir model, although acceptable non-linear adjustments were also obtained with Freundlich and Temkin models. These results indicate that Cr(VI) biosorption on TCP is a process where heterogeneous surface as well as monolayer biosorption might coexist. It has been determined a maximum Cr(VI) biosorption capacity *q*_max_ equal to 48.5 mg g^−1^, which is one of highest value among the non-treated agriculture waste biosorbents, reported in the literature. The adsorption kinetic data were well fitted with non-linear pseudo-2nd- and also with pseudo-1st-order models. These results would indicate that the Cr(VI) biosorption on TCP would be controlled by both, chemisorption and physisorption mechanisms. Intra particle diffusion model indicated a rapid diffusion of Cr(VI) ions from aqueous solution to outer surface of TCP and then a slow diffusion into its pores. However, both Weber-Morris and liquid diffusion models do not govern the rate-limiting step of the Cr(VI) biosorption. From thermodynamic point of view, the biosorption process is feasible and spontaneous (∆*G*^0^ < 0); endothermic (∆*H*^0^ > 0) with an increasing randomness (∆*S*^0^ > 0) at the solid–liquid interface. The desorption-regeneration experiments, using NaOH as the eluent, showed that TCP can be reused (%*D* > 40%) up to 3 times.

## Data Availability

The data that support the findings of this study are available from the corresponding author upon reasonable request.
